# Antiplasmodial Activity of a New Chemotype of *Croton sylvaticus* Hochst. Ex C. Krauss Essential Oil

**DOI:** 10.3390/ijms26020858

**Published:** 2025-01-20

**Authors:** Pierre Leonel K. Tafokeu Taguimjeu, Yannick Stéphane Fotsing Fongang, Manon Genva, Lahngong Methodius Shinyuy, Jana Held, Michel Frederich, Silvère Augustin Ngouela, Marie-Laure Fauconnier

**Affiliations:** 1 Laboratory of Chemistry of Natural Molecules, Gembloux Agro Bio-Tech, University of Liège, 5030 Gembloux, Belgium; pltaguimjeu@uliege.be (P.L.K.T.T.); m.genva@uliege.be (M.G.); 2 Department of Organic Chemistry, Faculty of Science, University of Yaoundé I, Yaoundé P.O. Box 812, Cameroon; sngouela@yahoo.fr; 3 Department of Chemistry, Higher Teachers’ Training College, The University of Maroua, Maroua P.O. Box 55, Cameroon; 4 Laboratory of Pharmacognosy, Center for Interdisciplinary Research on Medicine (CIRM), University of Liège, 4000 Liège, Belgium; ms.lahngong@doct.uliege.be (L.M.S.); m.frederich@uliege.be (M.F.); 5 Institute of Tropical Medicine, University of Tübingen, 72074 Tübingen, Germany; jana.held@unituebingen.de; 6 German Center for Infection Research, Partner Site Tübingen, 72074 Tuebingen, Germany

**Keywords:** *Croton sylvaticus*, essential oils, *Plasmodium falciparum*, antiplasmodial, gametocytes

## Abstract

*Croton sylvaticus*, a tropical African plant, is traditionally used to treat several diseases, including fever, inflammation, and malaria. Essential oils (EOs) from the plant’s leaves, roots, and trunk bark were obtained by hydrodistillation, and their chemical composition was analyzed by gas chromatography–mass spectrometry (GC-MS). The major constituents identified were virdiflorene (18.13 ± 0.46%) in root EO, (E)-β-caryophyllene (18.40 ± 0.60%) in trunk bark EO, and farnesyl acetone (15.26 ± 0.25%) in leaf EO. Notably, Cameroonian *C. sylvaticus* leaf EO exhibited a distinct and newly described chemotype with high levels of farnesyl acetone, β-copaene-4-α-ol, β-cadinene, α-humulene, and *trans*-longipinocarveol. In vitro testing revealed significant antiplasmodial activity against *Plasmodium falciparum* asexual (*Pf*3D7) and sexual (NF-54 strain) stages, with trunk bark EO showing the highest potency (IC_50_: 9.06 ± 2.15 µg/mL for *Pf*3D7 and 0.56 µg/mL for gametocytes). These findings support the traditional antimalarial use of *C. sylvaticus* and represent the first chemical profile and antiplasmodial efficacy report for its root and trunk bark EOs against both parasite stages. To the best of our knowledge, we also report for the first time the antiplasmodial activity of an EO that exerts significant activity against both the asexual and sexual forms of *P. falciparum*.

## 1. Introduction

Malaria remains one of the most dangerous and life-threatening parasitic diseases that is transmitted from person to person by an infected female *Anopheles* mosquito’s bite [1]. Of the five protozoan parasites responsible for malaria in humans, *P. falciparum* is the most aggressive, is the primary cause of severe malaria cases, and has led to the highest number of deaths in the World Health Organisation’s African Region [2,3]. In 2023, approximately 263 million cases of malaria accounted for approximately 597,000 deaths worldwide, with 95% of the cases occurring in Africa and 76% of deaths occurring in children under the age of 5 [4]. In Europe, approximately 6000 imported cases of malaria are recorded each year, of which 10% progress to severe malaria [4]. The life cycle of *P. falciparum* is extremely complex and involves several stages, occurring both in the human host and in the vector [5]. In humans, *P. falciparum* only undergoes the pre-erythrocytic phase in the liver, as it does not return to the liver after the erythrocytic phase. The exo-erythroctytic phase is carried out by other *Plasmodium* species. During asexual replication in the blood, however, a small fraction (less than 10% of the total asexual parasite population) differentiates to the transmission stages, referred to as gametocytes [3]. They differentiate through five different stages (Stage I to V), with mature Stage V male (micro-) and female (macro-) gametocytes being fully differentiated and representing the only stage of the parasite capable of transmission from the human host to the mosquito. Upon entering the mosquito, the micro- and macrogametes combine to create a diploid zygote, which subsequently transforms into a motile ookinete [3]. The ookinete penetrates the gut wall, forming oocysts that mature over time and ultimately release sporozoites. The sporozoites then travel to the mosquito’s salivary glands, where they are prepared to infect a human during a blood meal [6]. In the context of malaria elimination, it is crucial to focus on the discovery and development of new antimalarials that ideally demonstrate dual activity, being effective against both the asexual and sexual forms of the *P. falciparum* pathogen. This involves synergistic efforts to study different sources of drugs in search of potent compounds with antiplasmodial activities [3]. The prominent role of plant-derived natural products in antimalarial drug discovery [7,8] has prompted us to seek dual bioactive substances from *C. sylvaticus*, a medicinal plant used in folk medicine to treat malaria.

The genus *Croton*, belonging to the Euphorbiaceae family, includes approximately 1300 species of herbs, trees, shrubs, and sometimes lianas, predominantly found in warm tropical and temperate regions worldwide. Various *Croton* species have long been used in traditional medicine across Africa, South Asia, and South America to treat a range of conditions, including malaria, fever, dysentery, diabetes, cancers, digestive issues, wounds, inflammation, pain, and ulcers [9,10,11,12]. EOs extracted from these plants have also demonstrated biological activities such as antioxidant [13,14], anti-inflammatory, cytotoxic [14,15], antibacterial, antifungal [16,17], anti-parasitic [18], modulation of antibiotic [19], anxiolytic [20], and pharmacological effects [14]. *C. sylvaticus*, also known as the forest fever-berry, is a deciduous tree with abundant foliage and a broad, spreading crown. It can reach heights of up to 25 m and is commonly found in moist forests, dense thickets, and at the edges of forests. A comprehensive review of the literature shows that *C. sylvaticus* has traditionally been used to treat or manage a variety of ailments [21,22]. In Tanzania, an infusion of the leaves or roots is consumed orally to treat malaria [23,24], while in Kenya, stem bark infusions serve the same purpose [25]. In South Africa, decoctions made from the bark, leaves, or roots are used as traditional remedies for abdominal issues, swelling due to dropsy, fever, indigestion, internal inflammation, intestinal problems, rheumatism, and uterine conditions [26,27,28]. Previous studies indicate that *C. sylvaticus* exhibits a broad spectrum of pharmacological effects, including antiplasmodial, antifungal, antibacterial, larvicidal, and anti-inflammatory activities [8,11,24,29].

EOs are intricate blends of volatile compounds, typically characterized by low molecular weight, with terpenes, alcohols, and phenylpropanoids being the primary components [30,31,32]. Several EOs have been reported to possess antiplasmodial, antioxidant, anti-inflammatory, antimicrobial, antifungal, and antiviral activities [16,17,31]. Despite the large array of data available on EOs from other *Croton* species, knowledge about *C. sylvaticus* EOs remains scarce. The analysis of the EO obtained by hydrodistillation from the leaves of *C. sylvaticus* collected in South Africa revealed the presence of over fifty-two components, including sesquiterpenes/sesquiterpenoids, diterpenes/diterpenoids, and triterpenes/triterpenoids [33,34]. However, since the EO’s composition is heavily affected by a range of factors, including environmental ones [35,36], investigating *C. sylvaticus* from Cameroon could provide a new source of biologically active secondary metabolites. In this context, we investigated the chemical composition and the antiplasmodial activity of EOs obtained by hydrodistillation from the fresh leaves, roots, and trunk bark of *C. sylvaticus*. To our knowledge, no EOs from *Croton* species have been evaluated in vitro against the intra-erythrocytic asexual and sexual forms of *P. falciparum*. We also discuss the mode of action (MoA) of the active principles identified.

## 2. Results

### 2.1. Extraction of Essential Oils from C. sylvaticus

The hydrodistillation of fresh leaves, trunk bark, and roots from *C. sylvaticus* led to EO extraction yields of 0.70 ± 0.02% for the leaves, of 0.86 ± 0.05% for the trunk bark, and of 0.41 ± 0.08% for the roots.

### 2.2. Chemical Composition of Essential Oils from C. sylvaticus

The GC-MS analysis of EOs obtained from the different organs (leaves, roots, and trunk bark) of *C. sylvaticus* (Table 1) allowed for the identification of fourteen to eighteen compounds in these EOs, which mainly consisted of sesquiterpenes and diterpenoids. (E)-β-caryophyllene and virdiflorene were the major constituents in the trunk bark and root oil samples, while farnesyl acetone was the major constituent in the leaf oil. Other sesquiterpenes were also present in high proportions in all EO samples, such as caryophyllene oxide, α-humulene, δ-cadinene, β-cadinene, β-guaiene, spathulenol, and β-copaene-4α-ol. All samples contained higher proportions of sesquiterpenes compared with sesquiterpenoids. Diterpenes were also highlighted as significant components, with sclareol being one of the major components of EOs from the trunk bark and roots, and farnesyl acetone being one of the major components of EOs from the leaves.

#### 2.2.1. Root Essential Oil

**Figure 1:**
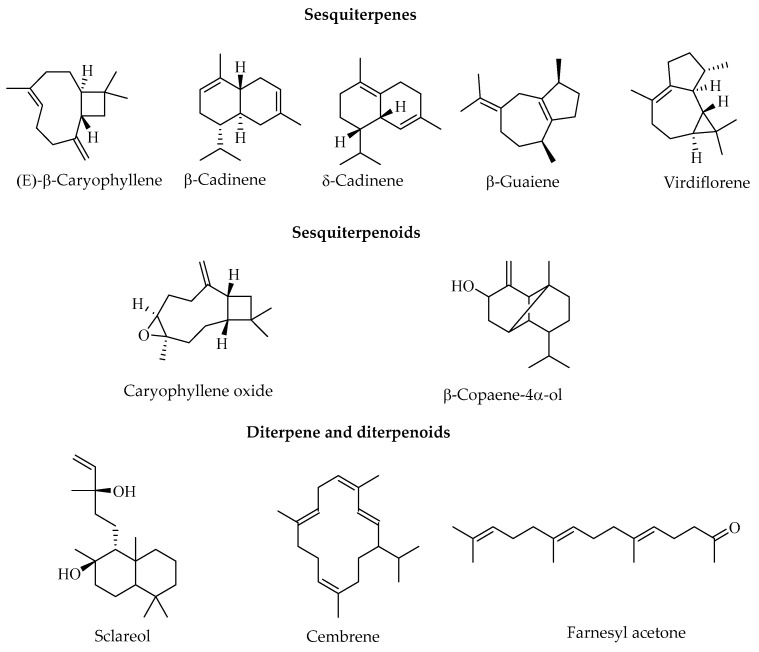
Main secondary metabolites identified in *C. sylvaticus* essential oils.

The GC-MS analysis of *C. sylvaticus* root EO allowed for the identification of seventeen compounds, including sesquiterpenes (53.04%), sesquiterpenoids (33.01%), monoterpenes (1.70%), monoterpenoids (1.73%), and diterpenoids (9.23%) (Table 1). The main molecules identified were virdiflorene (18.13 ± 0.46%), β-guaiene (15.38 ± 0.79%), spathulenol (9.58 ± 0.15%), and (E)-β-caryophyllene (6.86 ± 0.06%) (Figure 1). Monoterpenes included p-cymene (1.70 ± 0.12%) and isoborneol (1.73 ± 0.03%) (Figure 1). The only diterpenoid identified was sclareol (9.23 ± 0.27%) (Table 1).

#### 2.2.2. Trunk Bark Essential Oil

Eighteen compounds were identified in *C. sylvaticus* trunk bark EO, which mainly consists of sesquiterpenes (63.46%), including 41.84% sesquiterpene hydrocarbons and 21.62% oxygenated sesquiterpenes (Table 1). (E)-β-caryophyllene (18.40 ± 0.60%), caryophyllene oxide (12.61 ± 0.31%), α-humulene (8.54 ± 0.23%), and germacrene D (6.37 ± 0.24%) constituted the main sesquiterpenes. α-Pinene (2.27 ± 1.70%) represented the only monoterpene. Sclareol (15.75 ± 0.49%) and cembrene (12.86 ± 0.29%) were the main diterpenes in this oil (Table 1 and Figure 1).

#### 2.2.3. Leaf Essential Oil

GC-MS analysis of leaf EO allowed for the identification of fourteen compounds. The main constituents were sesquiterpenes (84.74%), including β-copaene-4-α-ol (14.23 ± 0.28%), δ-cadinene (12.57 ± 0.03%), β-cadinene (10.80 ± 0.09%), (E)-β-caryophyllene (9.64 ± 0.15%), α-humulene (8.40 ± 0.09%), and *trans*-longipinocarveol (7.62 ± 0.17%) (Table 1 and Figure 1). The only diterpenoid identified was farnesyl acetone (15.26 ± 0.25%) (Table 1 and Figure 1).

### 2.3. Evaluation of the Antiplasmodial Activities of Essential Oils from C. sylvaticus

#### 2.3.1. Inhibition of *P. falciparum* Asexual and Sexual-Blood Stages by Essential Oils from *C. sylvaticus*

Essentials oils hydrodistillated from the different organs (leaves, trunk bark, and roots) of *C. sylvaticus* were evaluated in vitro for their ability to inhibit the growth of a chloroquine-sensitive (*Pf*3D7) strain of *P. falciparum*. The results were categorized according to established classification criteria (IC_50_ ≤ 5 µg/mL: pronounced activity; 5 < IC_50_ ≤ 10 µg/mL: good activity; 10 < IC_50_ ≤ 20 µg/mL: moderate activity; 20 < IC_50_ ≤ 40 µg/mL: low activity; IC_50_ > 40 µg/mL: inactive), as outlined by Muganza et al. [37], with artemisinin serving as a positive control. Results highlight the good activities of the trunk bark EOs with an IC_50_ value of 9.06 ± 2.15 µg/mL, while the leaf and root oils exhibited very low activities with IC_50s_ values of 74.04 ± 28.87 and 71.46 ± 30.78 µg/mL, respectively (Table 2).

The root, trunk bark, and leaf EOs from *C. sylvaticus* were also tested in vitro for their impact on gametocyte viability. All the studied oils exhibited IC_50_ values of less than 2 µg/mL against gametocytes (NF-54 chloroquine-sensitive strain) of *P. falciparum*. Methylene blue, used as a positive control drug [3], had an in vitro gametocyte viability inhibition value of 85.11 µg/mL. All three tested essential oils therefore demonstrated very strong activity against the NF-54 strain of gametocytes. The trunk bark EO was the most potent, with an IC_50_ value of 0.56 µg/mL; this was closely followed by the essential oils of the leaves and roots, with IC_50_ values of 1.03 and 1.85 µg/mL, respectively (Table 2). This contrasts with the overall antiplasmodial activity against the sensitive *Pf*3D7 strain of *P. falciparum*, where the essential oils from leaves and roots showed very low activity.

#### 2.3.2. Inhibition of the Asexual Stage of *P. falciparum* in the Blood by Compounds from Trunk Bark Essential Oil

Overall, out of the eighteen compounds present in the essential oil of the trunk bark of *C. sylvaticus*, only three ((E)-β-caryophyllene, caryophyllene oxide, and sclareol) were tested due to their commercial availability and significant content in the extracted essential oils. Results (Table 2) show that all the compounds exhibited pronounced activity with IC_50_ values ranging from 0.48 to 1.30 µg/mL on the sensitive strain (*Pf*3D7) of *P. falciparum*. Interestingly, caryophyllene oxide was the most active one with an IC_50_ of 0.48 µg/mL.

#### 2.3.3. Cytotoxicity Assay

Due to its significant antiplasmodial activity, in vitro cytotoxicity tests were conducted exclusively on the EO from the trunk bark, using MDA-MB-231 cells (triple-negative breast cancer) to ensure that the observed activity was specific to the parasites. The level of toxicity was determined by calculating the selectivity index (SI) values, defined as the ratio of the half-maximal cytotoxic concentration (CC_50_) in MDA-MB-231 cells to the IC_50_ against *P. falciparum* (CC_50_/IC_50_ *Pf*3D7). The results indicate that the EO from the trunk bark was moderately cytotoxic, with a CC_50_ of 15.98 ± 0.86 µg/mL and a selectivity index of 1.76 (Table 2), indicating a low degree of differentiation between toxicity to host cells and inhibition of the studied parasitic strain.

## 3. Discussion

### 3.1. Extraction and Characterization of Essential Oils from C. sylvaticus

The EOs extracted from the fresh leaves, trunk bark, and roots of *C. sylvaticus* had yields of 0.70 ± 0.02%, 0.86 ± 0.05%, and 0.41 ± 0.08%, respectively. These values fall within the very broad yield range (0.02–6.41%) reported for *Croton* species [38,39]. Specifically, the EO yields from *C. sylvaticus* were similar to those of *C. jacobinensis* (0.70% for fresh stalks and 0.50% for leaves) and *C. regelianus* (0.50% for fresh leaves) [14], likely due to their comparable morphologies and chemical compositions.

The GC-MS analysis of the EOs of *C. sylvaticus* (leaves, roots, and trunk bark) revealed that sesquiterpene hydrocarbons were predominant in all the EOs. Indeed, these hydrocarbons are recognized for their broad spectrum of biological activities, including larvicidal [40], antiparasitic [41], antimicrobial, and antioxidant [42] activities. They are also used as hepatic protectors [43], anxiolytics, antidepressants [44], and neural protectors [45]. Therefore, their high content makes *C. sylvaticus* a very interesting species from an ethnopharmacological perspective.

Since the chemical composition and yield of EOs are influenced strongly by both internal genetic and developmental factors as well as external environmental ones [35,46], our study showed that the chemical composition of EO from leaves of *C. sylvaticus* harvested in Cameroon differs from that obtained from South Africa. This was evident from the presence of β-copaene-4-α-ol, δ-cadinene, β-cadinene, (E)-β-caryophyllene, α-humulene, and *trans*-longipinocarveol in the Cameroonian leaf EO, which were absent in the South African one [33]. Additionally, the main component in the South African oil, bicyclogermacrene, which makes up 81.8% [33], was not detected in the Cameroonian oil, while the primary component in the Cameroonian EO, farnesyl acetone (Table 1), was absent in the South African EO.

The findings do reveal sharp differences between the chemical profiles of the two oils. Variations in the composition of EO are indeed well documented among species from different geographic locations [47,48]. However, in cases where this variation is dramatic, it would be pertinent to refer to them as chemotypes, a term used to define plants of the same species that produce EOs with a distinct chemical composition and, hence, different biological activities [49]. Based on the above findings, our study identifies a new chemotype of *C. sylvaticus* leaf EO from Cameroon, characterized by a dominance of farnesyl acetone, β-copaene-4-α-ol, β-cadinene, and α-humulene. In contrast, the South African chemotype is primarily dominated by high concentrations of bicyclogermacrene, δ-cadinene, and β-bourbonene [33].

Future studies could leverage genetic and enzymatic analyses to enhance resource management strategies. Cultivating *C. sylvaticus*, native to South Africa, under Cameroonian conditions could offer valuable insights by enabling a direct comparison of essential oil compositions in standardized conditions.

Many studies have explored EO chemotypes from various plants. For example, common thyme (*Thymus vulgaris*) shows high polymorphism, with six distinct chemotypes featuring major compounds like thymol, geraniol, linalool, carvacrol, 4-thujanol/terpinen-4-ol, and borneol [47,49,50,51,52,53,54]. Another well-studied plant for EO polymorphism is tansy (*Tanacetum vulgare*). In Belgian tansy plants, the EO chemotype includes β-thujone, chrysanthenyl acetate, camphor, and thujone [55], whereas Norwegian tansy chemotypes contain thujone, chrysanthenyl acetate, camphor, chrysanthenol, and 1,8-cineole [56,57].

Our GC-MS analysis of *C. sylvaticus* essential oils (EOs) from leaves, trunk bark, and roots highlights the species’ chemical variability across different plant parts. In this study, the main compound in the roots was viridiflorene (Table 1), while (E)-β-caryophyllene and farnesyl acetone were predominant in the trunk bark and leaves, respectively (Table 1). Similar patterns of variation are seen in other plants. For example, in *Cinnamomum zeylanicum*, the EO from leaves is rich in eugenol [58], while the bark and roots are dominated by *trans*-cinnamaldehyde [59] and camphor [60]. Likewise, in *Lantana rhodesiensis*, EO composition varies between stems, leaves, and fruits [47].

Our study emphasizes the importance of rigorous chemical characterization of essential oils, as the substantial variability observed between sites and among different plant organs can lead to entirely distinct biological activities.

### 3.2. Antiplasmodial Activity and Cytotoxicity

The significant antiplasmodial activity of the EO from the trunk bark (IC_50_ = 9.06 ± 2.15 µg/mL) can be explained by its chemical composition, including sesquiterpenes (63.46%), diterpenes (32.91%), monoterpenes (2.27%), and a carboxylic ester (1.35%). In addition, the promising activity observed with this EO might be explained by individual constituents alone or by their combination (additive effect or synergy). For instance, Boyom et al. [61] showed that the strong antiplasmodial activity (IC_50_ = 2.0 µg/mL) of the essential oil from *Hexalobus crispiflorus* was attributed to its high sesquiterpene content (99.5%). Similarly, essential oil extracts from the bark of *Cleistopholis patens* and *Uvariastrum pierreanum* trunks harvested in Cameroon have shown strong antiplasmodial potential with IC_50_ values of 9.19 and 6.08 µg/mL, respectively [62]. These strong activities have been attributed to their high sesquiterpene content (˃81%). Indeed, sesquiterpenes are credited with various biological activities [63]. Additionally, research has demonstrated that sesquiterpenes are effective enhancers of skin penetration [62,64,65,66] and act as specific modulators of P-glycoprotein. They can help overcome cellular multidrug resistance by inhibiting the process of drug efflux [67]. In a previous study, oxygenated sesquiterpenes such as nerolidol and caryophyllene oxide (IC_50_ = 2.8 µg/mL) were identified as active ingredients against malaria parasites [68,69,70]. In our study, caryophyllene oxide (12.61 ± 0.31%) was found to be the most active compound in the EO of trunk bark, with an IC_50_ value of 0.48 µg/mL. It was followed by (E)-β-caryophyllene (18.40 ± 0.60%), with an IC_50_ value of 1.15 µg/mL. This difference in activity between these two sesquiterpene derivatives is due to the presence of the epoxide function. Indeed, the latter promotes interactions with the parasite’s enzymes, thereby disrupting the structure of the cell membranes and enhancing their ability to penetrate parasitic cells [71]. This reactive function thus plays a key role in the targeted toxicity against *P. falciparum*. Therefore, these compounds significantly contributed to the antiplasmodial activity demonstrated by the oil extracted from the bark.

In vivo studies have shown that sesquiterpenes disrupt the vital metabolic processes of the parasite and therefore prevent its proliferation [72]. Specific compounds, such as artemisinin, a sesquiterpene lactone derived from the plant *Artemisia annua*, have demonstrated exceptional efficacy against malaria [73,74,75]. Artemisinin and its derivatives are currently used as the standard treatment for malaria mainly in Africa where malaria remains a huge problem [4]. Thus, sesquiterpenes hold considerable potential as antimalarial agents due to their ability to target various aspects of *P. falciparum* biology. Their use in the development of new antimalarial treatments could provide effective and natural alternatives to current medications, especially in the context of growing resistance to traditional treatments.

Regarding diterpenes, the antiplasmodial potential of this class of compounds present in essential oils has garnered increasing interest in malaria treatment research. This activity has been observed both in vitro and in vivo, suggesting their potential as antimalarial agents. The study conducted by Koch, A et al. [76] reports that the abietane diterpene from *Fuerstia africana* showed very strong antiplasmodial potency with an IC_50_ of 1.95 µg/mL. In the paper published by Islam et al. [77], it is reported that diterpenes with one or more -OH groups may increase the membrane permeability of a variety of organisms, including *P. falciparum*. Because of their high lipophilicity, diterpenes can easily penetrate the cell membrane and create pores; thus, they can cause the loss of essential cellular components, shrinkage, and subsequent death. The diterpenes that have these properties include ent-beyer-15-en-19-ol (IC_50_ = 3.1 µg/mL) and sclareol [77]. In the specific case of the essential oil from the trunk bark of *C. sylvaticus*, kolavelool and sclareol (IC_50_ = 1.30 µg/mL) play a significant role in its antiplasmodial activity due to their structural diversity and lipophilicity. Furthermore, our study reports for the first time the specific diterpenes present in this EO.

Regarding gametocytocidal activity, the strong antiplasmodial potential of essential oils on gametocytes could be attributed to several compounds, notably (E)-β-caryophyllene, caryophyllene oxide, cembrene, and sclareol; however, terpene alcohols and ketones are not to be excluded. These compounds can act individually or in synergy to disrupt the development of gametocytes or inhibit their survival [78]. Indeed, they can induce alterations in the cell membrane of gametocytes, increasing permeability and causing the leakage of essential cellular constituents. This alteration of the cell membrane can also lead to acidification inside the parasite cells, blocking energy production and the synthesis of vital cellular components. Additionally, active components such as (E)-β-caryophyllene, caryophyllene oxide, and sclareol may interfere with the genetic material of gametocytes, causing irreversible damage that leads to cell death [3]. These combined actions could prevent the development and maturation of gametocytes, which is crucial for preventing malaria transmission via mosquitoes. These mechanisms, although effective in vitro, still require further studies to evaluate their efficacy in vivo and their safety for therapeutic application in humans.

Besides their biological functions, the physical attributes of essential oils, which include a low density (around 0.94 g/mL) and their capacity for easy diffusion through cell membranes, may improve the targeting of malaria parasites within cells [61].

The EO extracted from the trunk bark exhibited moderate cytotoxicity, with a CC_50_ of 15.98 ± 0.86 µg/mL and a low selectivity index (SI) of 1.76. An SI below 10 reflects limited antimalarial efficacy relative to its cytotoxicity, raising concerns about its safety and therapeutic potential [79]. The study conducted by García-Díez et al. [80] showed that the combination of thyme and cinnamon essential oils, selected for their strong antimicrobial activity, exhibits a significant synergistic effect against *Salmonella* spp., thereby suggesting the potential use of essential oil blends to control foodborne pathogens. Thus, to optimize the antiplasmodial properties of the EO from the bark, it would be relevant to combine it with the EO from the flowers of *Salvia verticillate*, known for its high caryophyllene oxide content (25.4%) [81]. Such a combination could produce a significant synergistic effect and thereby improve its selectivity index. Once proven safe, in vivo studies and clinical trials would be necessary to assess its efficacy in a clinical context.

In summary, specific compounds such as caryophyllene oxide (IC_50_ = 0.48 µg/mL), (E)-β-caryophyllene (IC_50_ = 1.15 µg/mL), and sclareol (IC_50_ = 1.30 µg/mL) are crucial for the dual antiplasmodial efficacy of the EO extracted from the trunk bark of *C. sylvaticus* (IC_50s_: 9.06 ± 2.15 µg/mL for *Pf*3D7 and 0.56 µg/mL for gametocytes). Based on the previous antiplasmodial results, these compounds represent good candidates for further studies, such as pharmacodynamics and pharmacokinetics studies, before in vivo validation of antimalarial efficacy using a malaria murine model. We believe that the strong antiplasmodial potency of these compounds is due to the presence of the epoxy group, their rigid bicyclic structure, and the phenolic group because these skeletons generate the production of free radicals, which leads to the death of the parasite. To our knowledge, we report for the first time the antiplasmodial activity of an EO that exerts significant activity against both the asexual and sexual forms of *P. falciparum*. This dual activity expands the therapeutic potential of this EO and opens new perspectives for the development of antimalarial treatments aimed at interrupting the transmission of malaria. This antiplasmodial potential not only supports its traditional use against malaria but also suggests that its chemical constituents could serve as raw materials for the development of an antimalarial drug.

## 4. Sustainability of *C. sylvaticus* Essential Oil

The exploitation of *C. sylvaticus*, a species whose EO, extracted from its trunk bark, has demonstrated antiplasmodial potential, raises important ecological and economic concerns. One of the main challenges is that bark extraction, although a source of active compounds such as caryophyllene oxide, (E)-β-caryophyllene, and sclareol, risks killing the tree by damaging its cambium, which is essential for sap circulation. These compounds, which have shown promising bioactive properties, could be used to develop treatments for malaria, further increasing their pharmaceutical value and the interest in their sustainable exploitation. To ensure the species’ sustainability, controlled cultivation through agroforestry or reforestation on degraded lands could, on the one hand, maintain viable tree populations and, on the other, provide income to local communities by sharing profits generated from the essential oil’s sale. *C. sylvaticus* may also exhibit a high natural regeneration capacity, as observed in similar species after major disturbances [82].

Simultaneously, alternative extraction methods beyond distillation that are less costly and more environmentally friendly, such as using volatile solvents, notably ethanol, or physical techniques (e.g., pressing, grinding), could be explored. Additionally, scientific research could diversify extraction sources by utilizing other parts of the tree, such as leaves, seeds, and/or fruits. By rethinking agricultural and extraction practices, it is possible to protect this species while creating a sustainable and environmentally friendly economic model. Moreover, ecological studies and monitoring programs provide critical data to guide conservation strategies, ensuring that this species remains a sustainable resource for future generations [83].

## 5. Materials and Methods

### 5.1. Chemicals and Reagents

Sclareol, (E)-β-caryophyllene, and caryophyllene oxide were obtained from Sigma-Aldrich (Darmstadt, Germany).

### 5.2. Plant Material

Fresh leaves, trunk bark, and roots of *C. sylvaticus* were collected in October 2016 on the slope of Ngoro Mountain (21°56′9.518″ N, 85°44′18.462″ E), department of Mbam-et-Kim, center region of Cameroon. The plant material was authenticated by Mr. Victor Nana, a seasoned retired botanist from the Cameroon National Herbarium (HNC), and its identity was cross-checked with a reference specimen cataloged under voucher number 11296SRF CAM. The plant materials were kept in a cold and dark room before being subjected to hydrodistillation.

### 5.3. Essential Oil Hydrodistillation

Fresh material (1000.0 g) of each part (leaves, roots, and trunk bark) was submitted to hydrodistillation for five hours using a Clevenger-type apparatus. The hydrodistillation was repeated thrice to determine EO yields. EOs were harvested, dried over anhydrous sodium sulfate, and stored in sealed vials at 4 °C until analysis. The yield (%) of EO was determined by calculating the volume of oil extracted from the plant material, using the formula outlined by Zhang et al. [84]:

$$\mathrm{Essential}\;\mathrm{oil}\;\mathrm{yield}\;\left(\%\right)=\frac{\mathrm{Essential}\;\mathrm{oil}\;\mathrm{mass}\;\left(g\right)\;}{\mathrm{Fresh}\;\mathrm{material}\;\mathrm{mass}\;\left(g\right)}\times 100$$

### 5.4. Essential Oil Analysis, GC-MS, and Data Analysis

Ten milligrams of EO was dissolved in 10 mL of hexane and analyzed by GC-MS. An Agilent 7890B GC system paired with a 5977B mass selective detector (MSD) (Agilent, Santa Clara, CA, USA), equipped with an HP-5MS capillary column (5% phenyl-95% methyl siloxane, 30 m × 0.25 mm, × 0.25 µm), was used for the analysis of non-polar phases, while a DB-Wax column (30 m × 0.25 mm, × 0.25 µm) was used for the analysis of polar phases, with helium as the carrier gas (1.2 mL/min). The use of columns with different polarities (HP-5MS and DB-Wax) allowed us to maximize the separation of compounds, improve the reliability of identifications, and achieve a more comprehensive analysis of the samples. One microliter (1 µL) of EO solution in hexane was injected in splitless mode. Adapted from Nea et al. [47], the oven temperature program was increased from 50 °C (1 min) to 300 °C (5 min) at a rate of 5 °C/min. The MSD operated at an ionization energy of 70 eV, covering a mass range from 40 to 400 atomic mass units. The source and quadrupole temperatures were maintained at 230 °C and 150 °C, respectively. Data analysis was conducted using MassHunter Workstation Software (Version B.08.00), specifically the Qualitative Analysis Navigator and Qualitative Analysis Workflows (Agilent Technologies, Inc. 2016, Santa Clara, CA, USA). Individual components were identified based on their chromatographic retention index (RI) and verified through spectral comparison with the NIST 17 library. The RIs were determined experimentally using a series of *n*-alkanes ranging from C_7_ to C_30_ and cross-referenced with published values in the literature [47,85]. The quantification of each component in each of the oils is expressed in relative peak area, each corresponding to the average of three independent repetitions.

### 5.5. Biological Assay

#### 5.5.1. In Vitro Inhibition of *P. falciparum* Asexual-Blood Stages

Asexual-blood stages of the chloroquine-sensitive 3D7 (*Pf*3D7) strain of *P. falciparum* were continuously maintained in vitro using RPMI1640 culture medium and used for antiplasmodial experiments. Briefly, the chloroquine *Pf*3D7 (MRA151) strain was obtained from the Malaria Research and Reference Reagent Resource Center, MR4, NIH (USA) and maintained in vitro as described by Trager and Jensen [86]. Briefly, parasites were maintained at 3% hematocrit (human type A or O-positive red blood cells) in a Complete Culture Medium (CCM) that was prepared by supplementing RPMI 1640 (Gibco, Fisher Scientific, Merelbeke, Belgium) medium containing NaHCO_3_ (32 mM), HEPES (25 mM), and *L*-glutamine with 10% heat-inactivated human plasma, 1.76 g/L of glucose (Sigma-Aldrich, Overijse, Belgium), 44 mg/mL of hypoxanthine (Sigma-Aldrich, Overijse, Belgium), and 100 mg/L of gentamycin (Gibco, Fisher Scientific, Merelbeke, Belgium). All cultures were placed in a humidified incubator at 37 °C with a standard gas mixture of 5% O_2_, 5% CO_2_, and 90% N_2_. Parasitemia was verified by microscopy using Giemsa-stained thin films.

A stock solution of EOs was prepared by dissolving 5 mg of extract in 1 mL of DMSO and diluting it with culture media. Each sample of EOs was tested in triplicate wells with the parasites at a final volume of 250 μL, making a final DMSO concentration of less than 1%. The positive control consisted of wells with parasites only while the negative control consisted of wells with culture medium only. A 1 μg/mL stock solution of artemisinin (Gibco, Fisher Scientific, Merelbeke, Belgium) was prepared and serially diluted as above and further used as a standard. After 48 h of incubation, parasite growth was estimated by determination of plasmodial lactate dehydrogenase activity as described by Kenmogne and collaborators [87], and IC_50_ values were calculated by linear regression. All tests of the EOs were repeated at least thrice, while the compounds were tested in a single replicate.

#### 5.5.2. In Vitro Assessment of the Gametocidal Activity

##### Induction of Gametocytogenesis and Maintenance of Parasite Cultures

Gametocytogenesis induction and gametocyte culturing were done as previously described [88]. Prior to initiation of gametocyte culture, synchronization of asexual-blood stages of the chloroquine-sensitive NF-54 *P. falciparum* strain was performed by D-sorbitol (5% *w/v*) and magnetic separation with LD-MACS. Synchronized ring-stage parasites were utilized to initiate gametocyte cultures (day 0) at a parasitemia of 0.3% and a hematocrit of 6% in a total volume of 25 mL using culture bottles (Nalgene 3110-42, Thermo Scientific, PA, USA). The complete culture medium, consisting of RPMI 1640 supplemented with 25 mM HEPES, 50 µg/mL hypoxanthine, 2 g/L NaHCO_3_, and 10% human serum, was entirely replaced every day for a duration of 14 days. On Day 3, when parasitemia reached 3% asexual stages, the hematocrit was lowered to 3%. To maintain a stable temperature of 37 °C, which is essential for gametocyte production and maturation, pre-warmed culture medium and a slide warmer (XH-2001, Premiere, C&A Scientific Co., Inc., Sterling, VA, USA) were employed. Under these conditions, the first gametocytes began to differentiate in culture by Day 6. The progression of sexual-stage development was observed microscopically using Giemsa-stained thin blood smears on Day 7, primarily showing asexual stages and Stage I to III gametocytes. At that time, cultures were treated from Day 13 to 14 (Stage IV and V gametocytes) with 50 mM N-acetyl-D-glucosamine to kill asexual parasites. On Day 14, Stage V gametocytes were purified with NycoPrep and MAC separation and a Neubauer cell chamber was used to count the number of gametocytes in the cell suspension and keep them in appropriate conditions for subsequent use.

##### Measurement of Gametocytocidal Activity

The ATP bioluminescence assay was used to determine the susceptibility of mature gametocytes to different extracts as described previously [87]. Stock solutions of extracts were prepared by dissolving 10 mg in 1 mL of 100% DMSO, and working concentrations were prepared by sterile dilution of the stock to at least 1:500 (DMSO < 0.4%) [87]. We used 50 µM Methylene-blue (the reference molecule for this test) prepared in sterile H_2_O as a gametocytocidal drug control. A total of 80 µL of medium was introduced into each well and 40 µL of the extract/drug (1:3 dilution) was added in well A and serially diluted to well G. The last wells were used as a growth control (well H). Additionally, a column for the gametocyte dilution control was equally prepared and stored at 4 °C until 30 min before use. A total of 20 µL of parasite suspension containing about 5000 gametocytes was added to each well except for the dilution control row (80 µL of parasite suspension to well A and 1:2 serial-diluted). Plates were incubated for 48 h at 37 °C with 5% CO_2_ and 5% O_2_. Following this incubation period, 30 μL of the BacTiter-Glo kit (G8231, Promega Corporation Madison, Wisconsin, USA) was added to measure the ATP levels of live parasites in accordance with the manufacturer’s guidelines. After adding the reagent (50 µL per well), luminescence was measured using a microplate reader (HTS counter Victor, Wallac, ID, USA), and the data were analyzed using R 4.3.2 software (R Development Core Team, Boston, USA) to determine the IC_50_ values. The assay was repeated at least twice.

#### 5.5.3. In Vitro Resazurin-Based Cytotoxicity Assay

The cytotoxicity of the essential oil from the bark of *C. sylvaticus* was assessed using the resazurin-based test [89] on MDA-MB-231 cells (triple-negative breast cancer). These cells were cultured in Dulbecco’s Modified Eagle Medium (DMEM) supplemented with 10% fetal bovine serum (FBS) and maintained in an incubator at 37 °C with a humidified atmosphere containing 5% CO₂. The MDA-MB-231 cells were then seeded in 96-well cell culture plates at a density of 2500 cells per well (2.5 × 10⁴ cells/mL) and were allowed to adhere and grow for 24 h before the treatments were applied. After this 24 h incubation period, the cells were exposed to the test samples in a complete medium (DMEM + 10% FBS), with DMSO concentrations adjusted to not exceed 0.5% in each well. The treatment duration was set at 72 h. Positive control wells received DMEM containing 0.5% DMSO, while negative control wells were treated with doxorubicin at a final concentration of 5 µM. At the end of the 72 h treatment period, cell viability was assessed by adding 10 µL of resazurin reagent solution (PrestoBlue, Thermo Fisher Scientific, ref. A13261, Waltham, MA, USA) to each well, according to the manufacturer’s instructions. After a 2 h incubation period at 37 °C under the same culture conditions, fluorescence was measured with a spectrofluorometer at an excitation wavelength of 560 nm and an emission wavelength of 590 nm. The fluorescence values obtained were then used to calculate cell viability relative to the controls. Results are expressed as 50% cytotoxic concentrations (CC50), and the selectivity index (CC50/IC50 *Pf*3D7) was calculated.

## 6. Conclusions

The aim of this work was to scientifically evaluate the relevance of the traditional medicinal use of the species *C. sylvaticus*, particularly in the treatment of malaria, through the characterization of its EOs and the evaluation of the antiplasmodial activity of different plant organs (leaves, roots, and trunk bark). Results show that sesquiterpenes were the predominant molecules in all samples. A new chemotype of EO of *C. sylvaticus* from Cameroon was established by our study and characterized by its high contents of farnesyl acetone, β-copaene-4-α-ol, β-cadinene, *trans*-longipinocarveol, and α-humulene. This chemotype differs from that of *C. sylvaticus* from South Africa, which is characterized by bicyclogermacrene, δ-cadinene, and β-bourbonene. Results also highlight the strong antiplasmodial potency of EOs extracted from trunk bark against both the asexual (*Pf*3D7) and sexual (gametocyte) stages of *P. falciparum* parasites, along with a discussion of their modes of action. Moreover, caryophyllene oxide, (E)-β-caryophyllene, and sclareol, the major compounds of the EO from the trunk bark, showed a very strong antiplasmodial activity against the asexual form of the parasite. Based on these activities, EOs obtained by hydrodistillation from trunk bark of Cameroonian *C. sylvaticus* have a high potential for use in traditional medicine for the treatment of malaria. However, in vivo assays on these EOs, along with cytotoxicity and gametocyte tests on their bioactive compounds and a study of their modes of action, are necessary to complete our study.

## Figures and Tables

**Table 1:** Composition of essential oils hydrodistillated from the leaves, trunk bark, and roots of *C. sylvaticus* (*n* = 3).

N°	Compounds	N° CAS	RIs				Rel. Prop. ^d^ (% ± SD)			Identification ^c^
			Non-Polar Column ^a^		Polar Column ^b^		Plant Parts			
			Exp.	Lit. ^e,f^	Exp.	Lit. ^e,f^	Leaves	Trunk Bark	Roots	
1	p-Cymene	99-87-6	1055	1048	1343	1322	-	-	1.70 ± 0.12	MS, RI ^i,j^
2	α-Copaene	3856-25-5	1379	1379	1529	1522	3.48 ± 0.07	-	-	MS, RI ^i,j^
3	α-Pinene	80-56-8	937	936	1187	1050	^_^	2.27 ± 1.70	-	MS, RI ^i,j^
4	β-Elemene	515-13-9	1396	1396	1610	1606	5.99 ± 0.17	-	-	MS, RI ^i,j^
5	Isoborneol	124-76-5	1164	1164	1692	1672	-	-	1.73 ± 0.03	MS, RI ^i,j^
6	Cyperene	2387-78-2	1402	1402	1552	1562	-	-	3.03 ± 0.03	MS, RI ^i,j^
7	Episesquithujene	15,9407-35-9	1395	1391	1569		-	3.0 ± 0.90	-	MS, RI ^i^
8	(E)-β-Caryophyllene	87-44-5	1422	1422	1609	1608	9.64 ± 0.15	18.40 ± 0.60	6.86 ± 0.06	MS, RI ^i,j^
9	(E)-α-Bergamotene	13,474-59-4	1442	1442	1607	1585	-	3.89 ± 0.34	-	MS, RI ^i,j^
10	α-Humulene	6753-98-6	1457	1457	1665	1665	8.40 ± 0.09	8.54 ± 0.23	2.49 ± 0.07	MS, RI ^i,j^
11	Germacrene D	23,986-74-5	1489	1489			-	6.37 ± 0.24	-	MS, RI ^i^
12	cis-5-Decen-1-yl acetate	67,446-07-5	1504	1591	1797		-	1.35 ± 0.06	-	MS, RI ^i^
13	Caryophyllene oxide	1139-30-6	1594	1594	1947	1955	-	12.61 ± 0.31	-	MS, RI ^i,j^
14	Epizonarene	41,702-63-0	1529	1530			2.35 ± 0.06	-	-	MS, RI ^i^
15	β-Copaene-4α-ol	124,753-76-0	1592	1591	2020	2135	14.23 ± 0.28	-	-	MS, RI ^i,j^
16	β-Selinene	17,066-67-0	1513	1509	1705	1702	3.45 ± 0.09	-	-	MS, RI ^i,j^
17	γ-Selinene	515-17-3	1478	1477	1708	1676	2.02 ± 0.22	-	5.34 ± 0.41	MS, RI ^i,j^
18	β-Guaiene	88-84-6	1498	1499	1566		2.47 ± 0.15	-	15.38 ± 0.79	MS, RI ^i^
19	β-Vetivenene	27,840-40-0	1522	1527	1777	1885	-	-	3.19 ± 0.41	MS, RI ^i,j^
20	β-Cadinene	523-47-7	1524	1523	1701	1692	10.80 ± 0.09	-		MS, RI ^i,j^
21	δ-Cadinene	483-76-1	1527	1527	1696	1708	12.57 ± 0.03	1.64 ± 0.05	4.45 ± 2.50	MS, RI ^i,j^
22	Virdiflorene	21,747-46-6	1533	1534	2113	1719	-	-	18.13 ± 0.46	MS, RI ^i,j^
23	Isolongifolan-8-ol	1139-08-8	1536	1531	1756		-	-	2.65 ± 0.40	MS, RI ^i^
24	Germacrene B	15,423-57-1	1553	1553	1981	1806	-	-	3.15 ± 0.09	MS, RI ^i,j^
25	Spathulenol	6750-60-3	1589	1589	1809		-	-	9.58 ± 0.15	MS, RI ^i^
26	τ-Muurolol	19,912-62-0	1664	1662	2089	2160	-	1.27 ± 0.49	-	MS, RI ^i,j^
27	Neointermedeol	5945-72-2	1670	1669			-	1.17 ± 0.40	-	MS, RI ^i^
28	Humulene epoxide II	19,888-34-7	1620	1620			-	4.29 ± 0.16	-	MS, RI ^i^
29	Caryophylladienol II	19,431-79-9	1681	1678	2136		-	2.28 ± 0.15	-	MS, RI ^i^
30	*Trans*-longipinocarveol	1,000,159-36-5	1615	1618	2241		7.62 ± 0.17	-	2.65 ± 0.18	MS, RI ^i^
31	Selina-3,7(11) -diene	6813-21-4	1666	1550	2090		-	-	6.32 ± 0.43	MS, RI ^i^
32	Khusimyl methyl ether	300,349-20-6	1690	1698	2123		-	-	2.83 ± 0.40	MS, RI ^i^
33	15,16-Dinorlab-12-ene, 8,13-epoxy-	5153-92-4	1896	1894	2096		-	1.62 ± 0.04	-	MS, RI ^i^
34	Cembrene	1898-13-1	1946	1948	2055	2180		12.86 ± 0.29		MS, RI ^i, j^
35	m-Camphorene	20,016-73-3	1958	1960	2074		-	1.30 ± 0.01	-	MS, RI ^i^
36	Kolavelool	19,941-81-2	1977	2079	2646		-	1.38 ± 0.06	-	MS, RI ^i^
37	Sclareol	515-03-7	2187	2198	2537		-	15.75 ± 0.49	9.23 ± 0.27	MS, RI ^i^
38	Ylangenal	41,610-68-8	1687	1674	2038		1.72 ± 0.43	-	-	MS, RI ^i^
39	Farnesyl acetone	1117-52-8	1926	1927	2202		15.26 ± 0.25	-	-	MS, RI ^i^
	Hydrocarbons monoterpenes (%)						//	2.27	1.70	
	Oxygenated monoterpenes (%)						//	//	1.73	
	Hydrocarbons sesquiterpenes (%)						61.17	41.84	53.04	
	Oxygenated sesquiterpenes (%)						23.57	21.62	33.01	
	Diterpenoids (%)						15.26	18.75	9.23	
	Hydrocarbons diterpenes (%)						//	14.16	//	
	Carboxylic ester (%)						//	1.35	//	
	Identified compounds (%)						˃99.99	˃99.99	98.71	

^a^ Database Retention Indexes (RIs) shown in this table for the non-polar column are the closest value to experimental data. ^b^ Database RIs shown in this table for the polar column are the closest value to experimental data. ^c^ Identification with the RI is reported with the specific column polarity. ^d^ Relative peak area at 1%. ^e^ Data from NIST 2023. ^f^ Data from Pherobase. ^i^ RI on the non-polar column. ^j^ RI on the polar column.

**Table 2:** Results of the antiplasmodial screening of EOs and selected pure compounds against asexual and sexual stages of the *P. falciparum* parasites and evaluation of their cytotoxicity (“-“ = not determined).

Organs	Antiplasmodial Activity *Pf*3D7/IC_50_ ± SD (µg/mL)	Gametocytes in Strain *Pf*NF-54/IC_50_ (µg/mL)	Cytotoxicity CC_50_ ± SD (µg/mL)	Selectivity Index (SI)
Roots	71.46 ± 30.78	1.85	-	-
Trunk bark	9.06 ± 2.15	0.56	15.98 ± 0.86	1.76
Leaves	74.04 ± 28.87	1.03 ± 0.73	-	-
Compounds (*n* = 1)				
(E)-β-Caryophyllene	1.15	-	-	-
Caryophyllene oxide	0.48	-	-	-
Sclareol	1.30	-	-	-
Artemisinin (µM)	0.03 ± 0.00	-	-	-
Methylene Blue	-	85.11	-	-

## Data Availability

All data generated for this study are included in the article/Supplementary materials.

## Supplementary Materials

The following supporting information can be downloaded at: www.mdpi.com/article/10.3390/ijms26020858/s1.
