# Supplementary material for: Antiplasmodial Activity of a New Chemotype of Croton sylvaticus Hochst. Ex C. Krauss Essential Oil
Source: Int J Mol Sci. 2025 Jan 20;26(2):858. doi: 10.3390/ijms26020858 (PMC11765546; doi:10.3390/ijms26020858)
Supplement: Supplementary file 1 [file ijms-26-00858-s001.zip › ijms-3384909-supplementary.pdf]

Antiplasmodial activity of a new chemotype of *Croton sylvaticus* Hochst. Ex C. Krauss essential oil

Pierre Leonel K. Tafokeu Taguimjeu [1,2], Yannick Stéphane Fotsing Fongang [3,*], Manon Genva [1], Lahngong Methodius Shinyuy [4,5], Jana Held [5,6], Michel Frederich [4], Silvère Augustin Ngouela [2] and Marie-Laure Fauconnier [1,*]

[1] Laboratory of Chemistry of Natural Molecules, Gembloux Agro Bio-Tech, University of Liège, 5030 Gembloux, Belgium (pltaguimjeu@uliege.be ; M.Genva@uliege.be ; marie-laure.fauconnier@uliege.be)

[2] Department of Organic Chemistry, Faculty of Science, University of Yaoundé I, P.O. Box 812, Yaoundé, Cameroon (sngouela@yahoo.fr)

[3] Department of Chemistry, Higher Teachers' Training College, The University of Maroua, P.O. Box 55 Maroua, Cameroon (fongangfys@yahoo.fr)

[4] Laboratory of Pharmacognosy, Center for Interdisciplinary Research on Medicine (CIRM), University of Liège, 4000 Liège, Belgium (ms.lahngong@doct.uliege.be ; m.frederich@uliege.be)

[5] Institute of Tropical Medicine, University of Tübingen, 72074 Tübingen, Germany (Jana.Held@uni-tuebingen.de)

[6] German Center for Infection Research, Partner Site Tübingen, 72074 Tuebingen, Germany

***Correspondence: YSFF (fongangfys@yahoo.fr; Tel: (+237) 696 13 28 33; ORCID 0000-0002-9311-9945)** and **MLF (marie-laure.fauconnier@uliege.be; Tel: (+32) 81 62 22 89)**

Table S1a: Composition of essential oils hydrodistillated from the roots of *C. sylvaticus* (n=3)

N°	Compounds	N° CAS	RIs				Identification [c]	Rel.Prop. [d] [%±SD]
			Non-polar column [a]		Polar column [b]			
			Exp.	Lit. [e, f]	Exp.	Lit. [e, f]		
**1**	p-Cymene	99-87-6	1055	1048	1343	1322	MS, RI [i, j]	1.70 ± 0.12
**2**	Isoborneol	124-76-5	1164	1164	1692	1672	MS, RI [i, j]	1.73 ± 0.03
**3**	Cyperene	2387-78-2	1402	1402	1552	1562	MS, RI [i, j]	3.03 ± 0.03
**4**	(E)-β-Caryophyllene	87-44-5	1422	1422	1609	1608	MS, RI [i, j]	6.86 ± 0.06
**5**	α-Humulene	6753-98-6	1457	1457	1665	1665	MS, RI [i, j]	2.49 ± 0.07
**6**	γ-Selinene	515-17-3	1478	1477	1708	1676	MS, RI [i, j]	5.34 ± 0.41
**7**	β-Guaiene	88-84-6	1498	1499	1566		MS, RI [i]	15.38 ± 0.79
**8**	β-Vetivenene	27840-40-0	1522	1527	1777	1885	MS, RI [i, j]	3.19 ± 0.41
**9**	δ-Cadinene	483-76-1	1527	1527	1696	1708	MS, RI [i, j]	4.45 ± 2.50
**10**	Virdiflorene	21747-46-6	1533	1534	2113	1719	MS, RI [i, j]	18.13 ± 0.46
**11**	Isolongifolan-8-ol	1139-08-8	1536	1531	1756		MS, RI [i]	2.65 ± 0.40
**12**	Germacrene B	15423-57-1	1553	1553	1981	1806	MS, RI [i, j]	3.15 ± 0.09
**13**	Spathulenol	6750-60-3	1589	1589	1809		MS, RI [i]	9.58 ± 0.15
14	*Trans*-longipinocarveol	1000159-36-5	1615	1618	2241		MS, RI [i]	2.65 ± 0.18
15	Selina-3,7(11) -diene	6813-21-4	1666	1550	2090		MS, RI [i]	6.32 ± 0.43
16	Khusimyl methyl ether	300349-20-6	1690	1698	2123		MS, RI [i]	2.83 ± 0.40
17	Sclareol	515-03-7	2187	2198	2537		MS, RI [i]	9.23 ± 0.27
	**Hydrocarbons monoterpenes (%)**							**1.70**
	**Oxygenated monoterpenes (%)**							**1.73**
	**Hydrocarbons sesquiterpenes (%)**							**53.04**
	**Oxygenated sesquiterpenes (%)**							**33.01**
	**Diterpenoids (%)**							**9.23**
	**Identified compounds (%)**							**98.71**

Table S1b: Composition of essential oils hydrodistillated from the trunk bark of *C. sylvaticus* (n=3)

N°	Compounds	N° CAS	RIs				Identification [c]	Rel.Prop. [d] [%±SD]
			Non-polar column [a]		Polar column [b]			
			Exp.	Lit. [e, f]	Exp.	Lit. [e, f]		
**1**	α-Pinene	80-56-8	937	936	1187	1050	MS, RI [i, j]	2.27 ± 1.70
**2**	Episesquithujene	159407-35-9	1395	1391	1569		MS, RI [i]	3.0 ± 0.90
**3**	(E)-β-Caryophyllene	87-44-5	1428	1428	1613	1613	MS, RI [i, j]	18.40 ± 0.60
**4**	(E)-α-Bergamotene	13474-59-4	1442	1442	1607	1585	MS, RI [i, j]	3.89 ± 0.34
**5**	α-Humulene	6753-98-6	1462	1462	1668	1670	MS, RI [i, j]	8.54 ± 0.23
**6**	Germacrene D	23986-74-5	1489	1489			MS, RI [i]	6.37 ± 0.24
**7**	cis-5-Decen-1-yl acetate	67446-07-5	1504	1591	1797		MS, RI [i]	1.35 ± 0.06
**8**	δ-Cadinene	483-76-1	1530	1530	1737	1737	MS, RI [i, j]	1.64 ± 0.05
**9**	Caryophyllene oxide	1139-30-6	1594	1594	1947	1955	MS, RI [i, j]	12.61 ± 0.31
**10**	Humulene epoxide II	19888-34-7	1620	1620			MS, RI [i]	4.29 ± 0.16
**11**	τ-Muurolol	19912-62-0	1664	1662	2089	2160	MS, RI [i, j]	1.27 ± 0.49
**12**	Neointermedeol	5945-72-2	1670	1669			MS, RI [i]	1.17 ± 0.40
**13**	Caryophylladienol II	19431-79-9	1681	1678	2136		MS, RI [i]	2.28 ± 0.15
**14**	15,16-Dinorlab-12-ene, 8,13-epoxy-	5153-92-4	1896	1894	2096		MS, RI [i]	1.62 ± 0.04
**15**	Cembrene	1898-13-1	1946	1948	2055	2180	MS, RI [i, j]	12.86 ± 0.29
**16**	m-Camphorene	20016-73-3	1958	1960	2074		MS, RI [i]	1.30 ± 0.01
**17**	Kolavelool	19941-81-2	1977	2079	2646		MS, RI [i]	1.38 ± 0.06
**18**	Sclareol	515-03-7	2195	2198	2537		MS, RI [i]	15.75 ± 0.49
	**Hydrocarbons monoterpenes (%)**							**2.27**
	**Oxygenated monoterpenes (%)**							**//**
	**Hydrocarbons sesquiterpenes (%)**							**41.84**
	**Oxygenated sesquiterpenes (%)**							**21.62**
	**Hydrocarbons diterpenes (%)**							**14.16**
	**Oxygenated diterpenes (%)**							**18.75**
	**Carboxylic ester (%)**							**1.35**
	**Identified compounds (%)**							**> 99.99**

Table S_{1c}: Composition of essential oils hydrodistillated from the leaves of *C. sylvaticus* (n=3)

N°	Compounds	N° CAS	RIs				Identification [c]	Rel.Prop.[d] [%±SD]
			Non-polar column [a]		Polar column [b]			
			Exp.	Lit. [e, f]	Exp.	Lit. [e, f]		
**1**	α-Copaene	3856-25-5	1379	1379	1529	1522	MS, RI [i, j]	3.48 ± 0.07
**2**	β-Elemene	515-13-9	1396	1396	1610	1606	MS, RI [i, j]	5.99 ± 0.17
**3**	(E)-β-Caryophyllene	87-44-5	1426	1426	1614	1613	MS, RI [i, j]	9.64 ± 0.15
**4**	α-Humulene	6753-98-6	1459	1459	1668	1668	MS, RI [i, j]	8.40 ± 0.09
**5**	δ-Cadinene	483-76-1	1489	1493	1739	1738	MS, RI [i, j]	12.57 ± 0.03
**6**	β-Guaiene	88-84-6	1501	1500	1565		MS, RI [i]	2.47 ± 0.15
**7**	β-Selinene	17066-67-0	1513	1509	1705	1702	MS, RI [i, j]	3.45 ± 0.09
**8**	β-Cadinene	523-47-7	1524	1523	1701	1692	MS, RI [i, j]	10.80 ± 0.09
**9**	Epizonarene	41702-63-0	1529	1530			MS, RI [i]	2.35 ± 0.06
**10**	β-Copaene-4α-ol	124753-76-0	1592	1591	2020	2135	MS, RI [i, j]	14.23 ± 0.28
**11**	*Trans*-longipinocarveol	1000159-36-5	1620	1618	1900		MS, RI [i]	7.62 ± 0.17
**12**	γ-Selinene	515-17-3	1663	1532	1709	1676	MS, RI [i, j]	2.02 ± 0.22
**13**	Ylangenal	41610-68-8	1687	1674	2038		MS, RI [i]	1.72 ± 0.43
**14**	Farnesyl acetone	1117-52-8	1926	1927	2202		MS, RI [i]	15.26 ± 0.25
	**Hydrocarbons sesquiterpenes (%)**							**61.17**
	**Oxygenated sesquiterpenes (%)**							**23.57**
	**Hydrocarbons diterpenes (%)**							//
	**Oxygenated diterpenes (%)**							**15.26**
	**Identified compounds (%)**							**> 99.99**

[a] Database RIs (Retention Index) shown in this table for non-polar column are the closest value to experimental data. [b] Database RIs shown in this table for the polar column are the closest value to experimental data. [c] Identification with the RI is reported with the specific column polarity. [d] Relative peak area at 1%. [e] Data from NIST 2023. [f] Data from Pherobase. [I] RI on the non-polar column. [j] RI on the polar column.

Table S2: Results of the antiplasmodial screening of EOs and selected pure compounds against asexual and sexual stages of the *P. falciparum* parasites and evaluation of their cytotoxicity. ("- "= not determined)

Organs	Antiplasmodial activity *Pf*3D7 / IC_{50} ± SD (µg/mL)	Gametocytes in strain *Pf*NF-54 / IC_{50} (µg/mL)	Cytotoxicity CC_{50} ± SD (µg/mL)	Selectivity index (SI)
**Roots**	71.46 ± 30.78	1.85	-	-
**Trunk bark**	9.06 ± 2.15	0.56	15.98 ± 0.86	1.76
**Leaves**	74.04 ± 28.87	1.03 ± 0.73	-	-
		**Compounds (n=1)**		
**(E)-β-Caryophyllene**	1.15	-	-	-
**Caryophyllene oxide**	0.48	-	-	-
**Sclareol**	1.30	-	-	-
**Artemisinin (µM)**	0.03 ± 0.00	-	-	-
**Methylene Blue**	-	85.11	-	-
